# Liquid biopsy based HER2 amplification status in gastric cancer patients indicates clinical response

**DOI:** 10.1016/j.heliyon.2023.e21339

**Published:** 2023-11-02

**Authors:** Susanne Klein-Scory, Swetlana Ladigan-Badura, Thomas Mika, Berlinda Verdoodt, Andrea Tannapfel, Michael Pohl, Roland Schroers, Alexander Baraniskin

**Affiliations:** a Department of Medicine, Ruhr University Bochum, University Hospital Knappschaftskrankenhaus Bochum GmbH, Germany; b Department of Hematology, Oncology and Palliative Care, Evangelical Hospital Hamm gGmbH, Germany; c Department of Medicine, Institute of Pathology, Ruhr University Bochum, Germany

## Abstract

Gastric carcinomas are among the most common cancers in Germany, with approximately 18,000 new cases per year.

About 10 years ago, based on results of the Trastuzumab for gastric cancer (ToGA) trial, the addition of the monoclonal antibody trastuzumab to a platinum-fluoropyrimidine chemotherapy backbone became the standard-of-care 1st-line therapy for human epidermal growth factor receptor 2 (*HER2*)-positive gastric cancers. Only patients with primary *HER2* gene amplification benefit from this therapy. Thus, accurate *HER2* gene amplification detection is predictive and critical for therapy selection. As a gold standard the *HER2* status is currently determined in tumor tissue specimens using immune histochemistry and fluorescent in situ hybridisation. However, *HER2* amplification is detectable in only about 20 % of gastric carcinomas. The recent approval of an antibody-drug conjugate Trastuzumab deruxtecan (T-DXd) and the establishment of a new subgroup of *HER2*-low tumors due to the bystander effect associated with T-DXd increases the relevance of precise *HER2* diagnostics.

Aim of this analysis was to determine the *HER2* amplification status from circulating DNA fragments in blood using a *HER2* Copy Number Variation assay to establish a minimal invasive approach.

For the present study, a digital droplet PCR-based method was validated relative to established tissue-based methods. Furthermore and most importantly, the changes of *HER2* status during therapy were investigated in seven patients indicating that the changes of *HER2* status and number of *HER2* copies detected in blood can reflect on therapy efficiency and uncover treatment resistance.

## 1 Introduction

Gastric cancer belongs to the most frequent malignant tumors in Germany with 18000 cases per year (https://seer.cancer.gov/statfacts/html/stomach.html) and nearly 40 % of the patients are younger than 65 years and employed at diagnosis.

For about 10 years, standard-of-care treatment for *HER2*-positive advanced gastric cancer includes the application of anti-*HER2* antibodies (trastuzumab). Human epidermal growth factor receptor 2, also called ERBB2, encoded by ERBB2 on chromosome 17, belongs to the human epidermal growth factor receptor (EGFR) family, which has an extracellular domain (ECD), a transmembrane domain and an intracellular tyrosine kinase domain. Binding of special ligands to the ECD activates signal transduction pathways important for tumor cell differentiation, growth, apoptosis, migration. Since HER2 lacks ligand-binding activity, it dimerizes with EGFR family members, activating numerous downstream signaling pathways that leads to cell transformation [1,2].

Although non-small cell lung cancer, colon, biliary track cancer, urothelial cancer and pancreatic cancers overexpress HER2 protein and/or show gene amplification in diverse proportions of cases, is most prominent in breast and gastric cancers [3]. HER2 protein overexpression is associated with poorly prognosis leading to targeted drug development [4].

The specific *HER2* receptor inhibitors reduce tumor cell growth by blocking the oncogenic growth factor receptor *HER2*. The effectivity of anti-*HER2* drugs depends on the overexpression of the *HER2* receptor, mostly as a result of gene amplification. Anti-*HER2* receptor therapy is not recommended for *HER2*-negative gastric cancer [5].

The *HER2* status testing algorithm involves immunohistochemistry (IHC) followed by fluorescence in situ hybridization (FISH) for moderately *HER2* expressing IHC results (IHC2+) [6]. As an alternative to gold standard FISH technology, chromatogenic in situ hybridization (CISH) technology and its variants can be used. In 304 breast cancer specimens, matches of >96 % were measured between FISH and CISH [2,7].

Currently, about 20 % of gastric cancer patients are found to overexpress *HER2* with a high likelihood to benefit from treatment with an anti-HER2 antibody therapy [8].

The anti-*HER2* antibody trastuzumab is a humanized recombinant monoclonal antibody that specifically binds to *HER2* ECD (part IV), suppresses the intracellular signaling and also due to a reduction of *HER2* receptors expression. Resistance to trastuzumab appears to be due to tumor heterogeneity. Furthermore, cell surface proteins like mucins, reduce the interaction of *HER2* receptors with trastuzumab [9]. Finally, the drug's inhibitory effect is blocked. To overcome resistance problem, various drugs and treatments are developed such as the antibodies pertuzumab, zanidatamab, pargetuximab, tyrosine kinase inhibitors lapatinib and tucatinib as well as the antibody-drug conjugates (ADC) like trastuzumab emtansine and trastuzumab deruxtecan.

In this context, the new ADC trastuzumab deruxtecan is particularly worth mentioning. It consists of a humanized, monoclonal, anti-HER2 antibody bound to a topoisomerase I inhibitor via a peptide-based linker demonstrated an excellent clinical efficacy of trastuzumab deruxtecan (T-DXd) in *HER2*-positive advanced gastric adenocarcinoma [10,11]. T-DXd targets the same antigen as trastuzumab but demonstrates increased benefit by delivery of the cytotoxic payload. It is partly due to the bystander anti-tumor effect of T-DXd, which occurs when the cytotoxic payload is released into the tumor cells, diffuses across membranes (due to the high membrane permeability of the payload), and then gets into and destroys neighboring tumor cells [12,13]. This property is especially useful for tumors with heterogenous expression of the targeted antigen such as *HER2*-positive gastric cancer [14]. These data resulted in the establishment of a new subgroup of *HER2*-low tumors, which could be treated with HER2-targeted agents. *HER2*-low cohorts are defined as patients with *HER2* IHC 2+/FISH− and IHC 1+ disease.

The proportion of patients with *HER2*-low gastric cancer is estimated to range between 5 % and 19 % [10,15]. Results of the patients in exploratory cohorts of phase II DESTINY-Gastric01 study showed an overall response rate of 26.3 % (n = 5/19) and 9.5 % (n = 2/21) in these respective subgroups.

Furthermore, in contrast to breast cancer tissues, gastric tumor tissues are thought to be intratumorally more heterogenous, leading to a relatively low *HER2* detection rate of 20 % of the initially analyzed tumors. It is estimated that this intratumoral heterogeneity leads to false results in about 23 % of the cases [8,16,17].

In 2013, a sensitive approach to detect *HER2* gene amplifications was published for breast cancer and gastric cancer cells [[18], [19], [20]]. Here, the circulating tumor DNA (ctDNA) released into blood was used to analyze the gene amplification status by digital droplet PCR based method (ddPCR). Gene amplification is measured using the copy number of the *HER2* gene versus reference genes found in liquid biopsies [[21], [22], [23]]. Overall, the ddPCR technology is an excellent tool serving this task enabling specific and very fast measurement of the absolute amount of DNA copies [24]. Secondly, the ctDNA reflects the most up-to-date status of the tumor genome to overcome the intratumoral heterogeneity not visible in tissue analyses [25,26]. In gastric cancers, the concordance between IHC/FISH analyses of the tissue and ddPCR based amplification detection was about 91 % [23,27]. Wang et al. used NGS to determine the *HER2* amplification status and analyzed 56 samples. The relative low concordance rate of about 60 % between tissue and liquid biopsy specimens were shown. It is important to consider that sequencing methods are more expensive, require more ctDNA, and are more time-consuming compared to the ddPCR based method.

The aim of our study was the determination of *HER2* amplification status of gastric cancer patients in cell free DNA from plasma, in which blood samples were collected next to diagnoses and during treatment. The samples were analyzed retrospectively. For this purpose, ddPCR-based measurements were used and the concordance of the ddPCR based method was reevaluated in relation to the established methods in tumor tissue and respect to the established method from Shoda et al. [21,22]. Furthermore, the cut-off for the assessment of *HER2* status was validated with control samples from non-cancer patients.

Liquid biopsy samples from 20 gastric cancer patients, 12 with and 8 without *HER2* amplification, and samples from 22 non-cancer patients were analyzed. In total, more than 450 *HER2* analyses of 72 blood collection time points were performed.

## 2 Materials and methods

### 2.1 Patient recruitment and blood collection

All patients were treated in the university hospital Knappschaftskrankenhaus Bochum GmbH. Patients' informed written consent was obtained prior to sample collection, analysis and usage of clinical data. The ethical committee of the Ruhr University Bochum, Germany, approved collection and analyses of samples for this study (permission number: 16–5962, date 28/02/2017). Peripheral blood samples were collected in K2-EDTA Vacutainer tubes (Becton Dickenson). Subsequently, plasma samples were prepared within 4 h, by centrifugation and stored as described [28].

### 2.2 HER2 analyses of tissue specimens

The HER2 status of patients were determined using standard procedure of IHC and FISH according to the TOGA rules using tumor tissue samples in routine pathological practice. In brief, for IHC the antibody Clone CB11 (Leica/Novocastra) was used on Leica Bond III automated staining machines according to the instructions of the manufacturer. For FISH, the Zytolight SPEC ERBB2/CEN 17 Dual Color Probe (Zytovision, Berlin, Germany) was used (CEN17: centromere chromosome 17). FISH analysis was performed if samples were scored as IHC score 2+; HER2 amplification by FISH was defined as a HER2/CEN 17 ratio 2 or more.

### 2.3 Isolation of DNA

Standard plasma volumes for ctDNA isolation by QIAamp circulating nucleic acid kit (Qiagen, Hilden, Germany) were 3 ml for ddPCR following the manufacturer's protocol (Qiagen, Hilden, Germany). The ctDNA isolation was done as described [28]. The ctDNA was analyzed by Bioanalyzer 2100 DNA D1000 chips (Agilent Technologies Inc. Santa Clara, USA) according to manufacturer's instructions.

The genomic DNA of peripheral blood cells of the patients was additionally purified using Qiagen DSP Blood isolation kit for total nucleic acid isolation. Formalin-fixed and paraffin-embedded tissue sections derived from primary tumor tissues were used for DNA isolation by Promega MAXwell 16 FFPE Plus lev DNA purification kit (As1135) with the Promega Maxwell instrument AS2000 or by Promega Reliaprep kit (Promega GmbH, Mannheim, Germany) according to the procedures established for routine clinical use (Institute for Pathology of the Ruhr University Bochum, Germany). To exclude that the detected *HER2* amplification originated and got passed on from blood cells we isolated the cellular DNA from blood cells and compared the copy numbers to the values detected in plasma.

### 2.4 ddPCR assays, CNV calculation and determination of the cut-off level

*HER2* gene FAM-labeled detection assay purchased from Bio-Rad was performed in combination with HEX-labeled reference gene detection assays as described previously [20,23,29]. In brief, the CNV of the target gene HER2 was determined by calculation of the ratio of the HER2 concentration (copies/μl) to the reference concentration (copies/μl), times the number of copies of reference in the human genome. The concentration of HER2 was determined 4–6 times simultaneously in relation to the respective reference gene. Information about reference genes and details of the ddPCR assays are summarized in supplementary information (SI Figures S1-S3). The ddPCR approach was established using the gDNA from cell lines of CAPAN1, CFPAC and KATO III, all with *HER2* amplification and from cell line HCT116 as a cell line without *HER2* amplification as given by Cosmic database (COSMIC https://cancer.sanger.ac.uk/cosmic). A comparison of data and accuracy of ddPCR HER2 analysis was given in supplementary information (Figs. S1–S3). The ddPCR based CNV of *HER2* was detectable with a maximal coefficient of variation of 0.1 and an inter-assay variability of 0.3.

Contingency table were analyzed by Graph path prism 4 (9.5.1) using Fisher's exact test (alpha 0.5).

## 3 Results

### 3.1 Patients characteristics

The patients'characteristics were summarized in Table 1. We collected blood samples from 20 gastric cancer patients. Twelve patients were assessed as HER2 positive (overexpressed/amplified), of those 9 patients were scored as IHC3+ and 3 patients as IHC2+ with FISH amplification, and 8 patients as negative by tissue IHC and FISH. The dependency of the HER2 status from histological features of the tumor (as reported by Ma et al., 2023 [2]) was not evident in our small group of patients. The patients in the HER2 positive group have 58 % tubular, and 50 % intestinal typed (according to Laurén) adenocarcinoma, and patients in the HER2 negative group had 37 % tubular, and 37 % intestinal typed.

**Table 1:** Characteristics of gastric cancer patients.

ID	age	sex	tissue HER2 IHC score	Initiale TNM stage^a^	Grading	UICC	type Laurén	Histology adenocarcinoma	response to anti-HER2 chemotherapy
GC11	76	m	**3** + **pos**	cT3 cN0 M1 (pul)	G2	IIA	intestinal	moderately differentiated tubular	Responder, sec. resist
GC13	65	f	**3** + **pos**	cT3 N1 Mx (hep, splenic)	G2	IV	intestinal	moderately differentiated tubular	Responder, sec. resist
GC15	68	m	**3** + **pos**	ypT3 pN1pM1 (lym, per)	G3	IV	*na*^a^	poorly differentiated, Barrett CA	Responder, sec. resist
GC02	53	f	**2** + **pos,** **FISH** + **6.8**	uT3 N + M1 (bone metastasis)	G3	IV	diffuse	poorly differentiated, signet ring cellular	Responder, SD
GC20	56	m	**2** + **pos,** **FISH** + **6.6**	uT3 N+ cM0	G2	IIB	intestinal	moderately differentiated tubular	Responder, CR
GC21	60	m	**3** + **pos**	uT3 N2 M1 (pul, hep)	G2	IV	diffuse	AC of the gastroesophageal junction	Responder, sec. resist
GC22	74	m	**3** + **pos**	uTx Nx M1 (pul, hep)	G2	IV	*na*	moderately differentiated, gastroesophageal type	Responder, sec. resist
GC05	72	m	**3** + **pos**	uT3 μN + M0	na	IB	mixed	poorly differentiated	No trastr, SD
GC07	59	m	**3** + **pos**	uT3 μN1 μM1 (hep)	G2/3	IV	diffuse	poorly differentiated tubular,	Responder, sec. resist
GC08	63	m	**3** + **pos**	uT2 μN2 cM0	G2	IIIA	intestinal	well differentiated tubular	Responder, sec. resist
GC09	74	m	**3** + **pos**	cTx cN1 cM (hep, per, splenic)	G1	IV	intestinal	well differentiated tubular	Responder, sec. resist
GC19	64	m	**2** + **pos,** **FISH** + **2.3**	cT3 N0 M0	G3	IIA	*intestinal*	*poorly differentiated tubular*	*Primary resistant*
GC01	63	m	neg	uT3 μN + M0	G1	IIB	intestinal	well differentiated tubular	No trastruzumab
GC03	66	m	neg	uT3 μN + M0	G2	IIIB	*na*	AC of the lower third of the oesophagus (AEG)	No trastruzumab
GC04	32	f	neg	uT3 μN + M0	G3	IIB	diffuse	poorly differentiated, signet ring cellular	No trastruzumab
GC14	66	f	neg	uT3 μN + M0	G2	IIIB	*na*	moderately differentiated tubular AC of gastric antrum	No trastruzumab
GC16	58	m	neg	uT3+ μN + M0	G2	IIIA	*na*	moderately differentiated tubular	No trastruzumab
GC17	61	m	neg	uTx N0 M1 (hep)	G3	IIIC	diffuse	poorly differentiated	No trastruzumab
GC18	60	m	neg	uT3 μN + cM0	G3	IIB	intestinal	poorly differentiated	No trastruzumab
GC10	52	m	na	pT3 pN1 cM0 L1 V0 Pn1 R0	G3	IIIA	*na*	poorly differentiated, distal oesophageal type	No trastruzumab

a na, not assessed or data missing, HER2 amplification was assessed by FISH >2.4, IHC score according to TOGA criteria, *Hep - hepatic, pul - pulmonal, per – peritoneal, f – female, m – male, AC adenocarcinoma, responder, sec resist – secondary resistant. The non-cancer group of patients includes patients with gastrointestinal symptoms without signs of malignancy.

### 3.2 *HER2* amplification status in tissue: comparison of CNV detection methods IHC/FISH versus ddPCR

As no tissue was available from one patient, the comparison between IHC/FISH results and ddPCR CNV measurement was performed on 11 of 12 GC patients. The ddPCR-based copy number variation assay (CNV), described in Material & Methods section, can detect *HER2* gene amplification in 10 of 11 samples in genomic DNA from tumor tissue (Table 2). The one tCNV not consistent with the IHC3+ IHC result derived from a patient with an intestinally typed tubular adenocarcinoma. For this purpose, the cut-off of 2.4 established by Shoda et al., 2015 was used [21]. The results of ddPCR based *HER2* CNV detection using tissue gDNA (tCNV) were in 91 % agreement with the IHC/FISH analyses of the tissue material (p = 0.0006*, likelihood ratio 2.8). The Spearman's coefficients were summarized in Supplementary Fig. S4A. All reference genes had a strong concordance to each other (Spearman coeffient >0.88, supplementary file Fig. S4).

**Table 2:** ddPCR based CNV measurement: comparison tissue DNA versus ctDNA.

tissue gDNA Baseline					ctDNA first sample			
pat	Patho HER2 status (TOGA)	Mean tCNV^∗1^	tCNV Ref_Chr.17	gDNA HER2 status	T0 vs B0 (weeks)	Mean pCNV^∗^^^1^^	pCNV Ref_Chr.17	ctDNA HER2 status
GC11	Her2 pos	**1.75 ± 0.13**	**2.21**	**neg**	58	1.9 ± 0.3	2.26	neg
GC13	Her2 pos	3.96 ± 0.6	3.15	pos	30	2.2 ± 0.4	1.94	neg
GC15	Her2 pos	16.8 ± 4.7	16.3	pos	2	1.9 ± 0.4	1.53	neg
GC2	Her2 pos	11.5 ± 4.1	11.5	pos	**1**	**6.2 ± 1.3**	**7.4**	**pos**
GC20	Her2 pos	55.1 ± 12.3	33	pos	7	2.2 ± 0.1	2.38	neg
GC21	Her2 pos	12.1 ± 1.0	11.26	pos	3	1.9 ± 0.3	1.74	neg
GC22	Her2 pos	49 ± 5.5	23.4	pos	**0**	**30.3 ± 6.1**	**17.4**	**pos**
GC5	Her2 pos	2.9 ± 0.5	2.57	pos	5	2.0 ± 0.4	1.9	neg
GC7	Her2 pos	3.4 ± 0.6	2.35	pos*^2^	73	2.2 ± 0.6	1.8	neg
GC8	Her2 pos	4.7 ± 0.3	2.13	pos*^2^	60	2.1 ± 0.2	1.97	neg
GC9	Her2 pos	4.2 ± 1.4	5.2	pos	17	1.9 ± 0.3	1.74	neg
GC19	Her2 pos	na	na	No tissue	12	1.9 ± 0.4	1.8	neg

The ddPCR based analysis of the reference gene located near the *HER2* gene on chromosome 17 can successfully distinguish polysomy of chromosome 17 from *HER2* gene amplification (patients GC7 and GC8) (Table 2, all data are listed in the supplementary file 2).

Table 2 CNV of *HER2* gene were determined from specimens of patients with positive *HER2* status using ddPCR assays. **tCNV** - gDNA from tissue specimens, **pCNV** – ctDNA from plasma used in ddPCR CNV assays. Sensitivity of the ddPCR-based assay from tissue derived gDNA is 90 % with a specificity of 100 %, likelihood +2.8, p-value 0.0006 (Fisher's test alpha 0.05). If the blood was drawn simultaneously to the tissue sampling, *HER2* gene amplification can be detected in the ctDNA from blood. T0 vs B0 = time between tissue removal date T0 and first blood collection date B0. *^1^ddPCR based CNV-analysis *HER2* gene (± standard deviation) on chromosome 17 versus 4 different reference genes (EIF2C1 Chr1, RPP30 Chr10, RPPH1 Chr14 und TERT Chr5), *^2^ Comparison of CNV calculated by reference on chromosome 17 versus CNV using the reference genes outside chromosome 17 can indicate polysomy of chromosome 17.

#### 3.2.1 Results of CNV using circulating tumor DNA (pCNV)

**Fig. 1:**
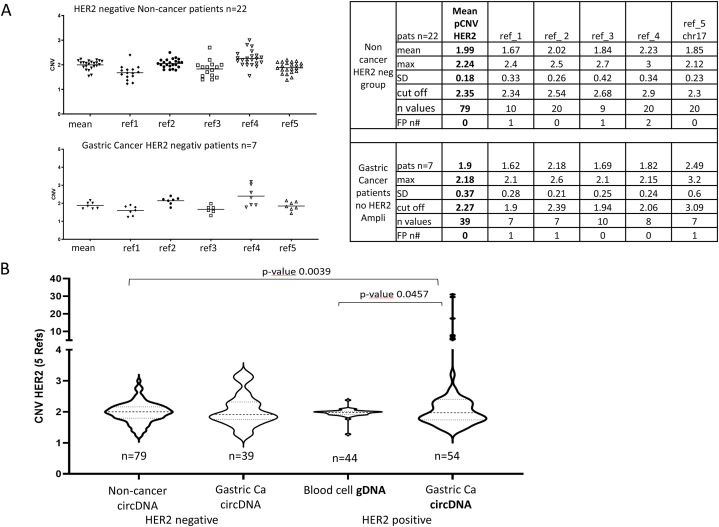
**Re-Validation of cut-off CNV depending on reference genes and patient groups**: **A** CNV is dependent on the reference gene used for CNV determination. The cut off at 2.4 given by Shoda et al., 2015 can be used for diagnosis if mean CNV values from 4 to 5 reference genes are used for HER2. CNV-HER2 values calculated with one reference gene, on the other hand, lead to false positive classifications. **B** CNV values per patient group determined with 5 reference genes were summarized by violin plots. Blood cell-derived genomic DNA from gastric cancer patients with HER2 amplification can be used as a negative control to classify ctDNA-based CNV values (for details see supplementary information). (SD – standard deviation, FP n# - number of false positive detected patients).

The cut-off value reported by Shoda et al., 2015 [21] was tested using samples from patients without malignant disease and from gastric cancer patients who were classified as *HER2*-negative based on tissue biopsy analysis (Fig. 1). The pCNV varied dependently from the reference gene used. If only one reference gene used for pCNV determination, some sample would be classified as HER2 amplified in the specimens of non-cancer people (supplementary file Fig. S4D).

In the small group analyzed herein, the cut off of 2.4 given by Shoda group can discriminate the groups without false positive results, if the results of 4–5 reference genes were used.

In order to exclude that changes of hematopoietic cells influence the pCNV, the cellular gDNA from blood cells of corresponding plasma samples was additionally measured by ddPCR from 6 HER2 positive patients. In the 6 specimens of HER2 positive patients, the cellular gDNA derived CNV was below the cut off of 2.4 (Supplementary Fig. S5), suggesting that the cellular gDNA can be used as an individual control to the pCNV measurement.

Due to the high individual variability of reference gene copies in patient samples, only the measurement of *HER2* gene copies in relation to several different reference genes can be used to determine the mean CNV. Narrowing down *HER2* CNV determination based on only 1–2 reference genes would lead to frequent false positive results (Fig. 1). The measurements of the patient collectives confirmed the established cut-off value of 2.4 in gastric carcinoma and the concordance values obtained in 60 patients by Shoda et al., 2017 (72–93 %) [22].

Thus, the ddPCR-based detection method for *HER2* amplification in gastric carcinoma from ctDNA in blood is qualified to monitor the changes during treatment.

The good agreement between IHC/FISH and tissue based CNV determination (tCNV) (Table 2) was not confirmed regarding the pCNV from specimens at the first sampling time. In two cases the *HER2* amplification can be accurately measured using circulating tumor DNA (ctDNA) from plasma, resulting in a sensitivity and specificity of 0.2 and 1, with an estimated likelihood ratio of 20 (p-value 0.48; supplementary file Fig. S4C). In one case the pCNV confirmed the negative tCNV result.

Blood samples of the two accurately detected cases (GC2 and GC22, Table 2) were obtained at nearly the same time (±1 day) as cancer tissue sampling. In all other cases the blood collection took place after the start of therapy or after surgery. The tissue of the patient GC2 was described as a signet-ring cell tumor of diffuse type according to Laurén with moderate HER2 expression (IHC2+) and HER2 amplification detected by FISH. The tissue of the 2nd pCNV HER2 positive patient showed high HER2 expression (IHC3+) and was typed as gastroesophageal moderate differentiated tumor tissue.

**Fig. 2:**
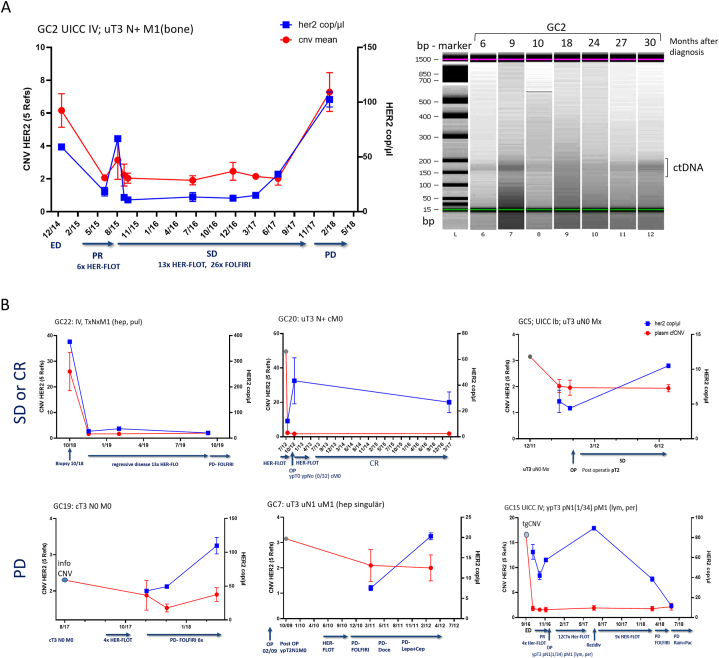
**HER2 CNV monitoring of gastric cancer patient during treatment with initial HER2 amplification.** Monitoring of HER2 CNV (red) from HER2 copy number relative to 4–5 reference gene copies and absolute amount of HER2 ctDNA (blue) were measured at different time points during therapy. **A** HER2 CNV and ctDNA HER2 cop/μl decrease with response to initial therapy in a stage IV patient. Response to therapy is reflected by the decrease in CNV and absolute levels of ctDNA. After a stable phase of disease, the ctDNA-HER2 amount and HER2-CNV increase again with disease progression (left). The ctDNA bioanalyzer show increased amount of ctDNA at 165 bp at the beginning of therapy and during progression (right). **B** HER2-CNV monitoring of 6 patients: HER2-CNV and HER2 ctDNA remain consistently low in patients with stable disease or complete remission; with relapse and progression, HER2 ctDNA levels increase. CR complete remission, PD progression disease, SD stable disease, ED diagnosis of gastric cancer.

In seven patients with tissue *HER2* positive status, pCNV was measured during therapy. After initiation of therapy, no amplification of the *HER2* gene was detectable in any patient sample (Table 2, Fig. 2). The amount of ctDNA was determined by absolute *HER2* gene copies measured in ddPCR and additionally by Bioanalyzer measurements (Fig. 2, Supplementary Fig. S6). The course of therapy is reflected in changes in *pCNV* and in the amount of *HER2* ctDNA in the blood (Fig. 2A). When the tumor burden is high during disease progression, both *HER2*-ctDNA levels and CNV levels increase. In patients with complete remission or stable disease, *HER2* ctDNA levels remain low and pCNV level remain constant (Fig. 2B).

This work is a preliminary experience based on a limited series of cases (summarized in Supplementary Fig. S7).

## 4 Discussion

Gastric cancer is a heterogenous group of diseases with different responsiveness to treatments and therapeutic decisions should consider the molecular tumor profile. The benefit of trastuzumab treatment is limited to gastric cancer with *HER2* overexpression status of IHC 3+ or moderate overexpression (IHC 2+) with *HER2* gene amplification (FISH positive) [30].

A main prerequisite for the accurate molecular characterization of the tumor is the selection of the optimal analysis method. The TOGA criteria summarizing the properties for detection of *HER2* amplifications in tissues are based on two technologies, FISH and IHC. Both analysis methods are time-consuming, resource-intensive. The chromatogenic in situ hybridization (CISH) technology and its variants was developed to reduce the costs. Both FISH and CISH are approved by FDA and gave concordant results in over 98 % of breast cancer specimens [7]. However, sometimes IHC/FISH analyses gave false results. The use of different fixatives and the storage time of specimens influenced the detectability of HER2 [31]. Particularly, for gastric cancer tumors with IHC 2+, the rate of *HER2* gene amplification in tumor tissue specimens was only 38–56 %, probably due to intratumoral heterogeneity. The discordance of *HER2* gene amplification results among surgically resected and biopsy specimens underscores this feature [[31], [32], [33], [34]].

Analysis of ctDNA from blood (liquid biopsies) instead of tissue specimens can be advantageous to prevent from false results. For liquid biopsy samples the ddPCR approach can be used to detect the *HER2* amplification [18,23,27]. In principle, liquid biopsy based HER2 detection can overcome the dependence on the quality of the tissue biopsy, which in turn is influenced both by the marked heterogeneity in gastric cancer and by the type of fixation and the age of the sample.

The preliminary data presented here, support a cut-off level of *HER2* CNV amplification at > 2.4, in particular, if it was used in relation to at least 4–5 reference genes. This value is relatively low for example in contrast to the CNV cut-off level estimated by whole genome sequencing approaches given with >4 [27,35]. These recently used methods to determine the CNV status of *HER2* need more resources as the ddPCR based technology. The ddPCR pCNV detection on 60 gastric cancer and 30 “healthy” patients had shown a sensitivity and specificity of 0.73 and 0.93 (p-value 0.001) using preoperative samples [22]. The data on 29 gastric cancer patients with preoperative corresponding liquid biopsy samples gave concordance between tCNV and pCNV of 96.6 % using a cut off of 2.4 [36].

The liquid biopsy based pCNV determination is very strongly dependent on the time of blood collection in relation to the therapy [22,31]. All three samples collected at baseline confirmed the tissue based CNV determination, and showed two HER2 positive and one HER2 negative results. 84 % of blood samples in the small group of cases presented here were collected after treatment initiation and had pCNV below the cut off of 2.4. The loss of HER2 positivity during treatment of gastric cancer was described also in tissue samples and it was associated with resistance to trastuzumab therapy regimen [31,34].

The potential of ddPCR based *HER2* amplification measurement from blood, as previously described for breast, colon, and gastric tumors, was also confirmed in the gastric cancer samples presented herein. The *HER2* amplification status may change due to clonal growth and intratumoral selection during the progression of the tumor. Newer approaches demonstrated that 54 % of treated gastric cancer patients altered the initially measured *HER2* status during therapy [22,32]. The systematic comparison of molecular analyses of tissue-derived versus liquid biopsy-derived DNA collected simultaneously from 23 patients revealed that molecular changes were found more frequently in liquid biopsy samples than in tissue biopsies, 87 % versus 48 % respectively [35]. In addition, diverse *HER2* expression in metastatic lesions and primary tumors [27,37] together with *HER2* expression levels changing during treatment, reinforce the need for testing subsequent to diagnosis during therapy and at disease progression.

The present data on therapy progression of gastric cancer patients show that, as in other tumor entities, tumor cells with *HER2* amplification are effectively reduced under targeted therapy. Tumor cells with *HER2* amplification are no longer measurable in liquid biopsy as a sign of efficient therapy. Conversely, disease progression can lead to a redetection of tumor cell DNA, i.e., detection of *HER2* amplification from blood. In particular, the approach can help to map the success of a (also neoadjuvant) therapy.

As a minimally invasive procedure the liquid biopsy based *HER2*-CNV detection method is more suitable to monitor the molecular changes during therapy as tissue biopsies. The liquid biopsy approach eliminates the need for hospitalization associated with side-effect-prone tissue removal. The procedure is risk-reducing for patients and particularly resource-saving due to the short time required for analysis (only one working day from ctDNA isolation to the result) and the relatively low material cost. The clinical application of the method, particularly for the evaluation of therapy success, is therefore desirable. Furthermore, there is a considerable socioeconomic aspect: since approximately 40 % of gastric cancer patients are younger than 65 years at the time of diagnosis and are thus capable of working, the non-invasive examination procedure preserves the ability to work of many of those affected and results in fewer costs due to absences from work.

In view of the more effective HER2-targeting therapies that are coming into clinical use like Trastuzumab deruxtecan (T-DXd) and the need to differentiate the new subgroup of HER2-low, there is an increasing need for more precise and sensitive HER2 testing methods to identify patients who might benefit from anti-HER2 targeted treatment.

## 5 Conclusion

The data presented here may serve as a basis for clinical studies that help to assess a prognostic or predictive value of the analytical procedure in particular in respect to the new treatment options like T-Dxd which was approved from FDA for treatment of advanced HER2-positive gastric cancer in January 2021. In breast cancer, HER2-low expressing tumors represent a subgroup benefiting from T-Dxd. The definition of the HER2-low status includes either tumors with an IHC score 1+ or with an IHC score 2+ but without HER2 amplification evidence. The liquid biopsy based ddPCR approach with its robustness and performer independency can be advantageous to define the subgroup of HER2-low expressing gastric tumors.

Studies analyzing HER2 expression ctDNA analyses will help to enlighten the connection between HER2 status and for example T-DXd efficacy and the bystander antitumor effect, probably resulting in more accurate patient selection and precise prediction of response.

Basically, the determination of HER2 positivity is one of the crucial challenges considering the development of HER2-targeted therapies for gastric cancer. Importantly, the semi-quantitative determination of HER2 expression in IHC and the HER2 amplification detection, could not provide a dichotomous distribution of HER2 status, but rather there is a continuous spectrum with cut-off point to be determined. The non-invasive determination of the current valid HER2 status via liquid biopsy would significantly increase the specificity of the subgroup definition of patients for different anti-HER2 drug and would thus represent a milestone in the development of targeted therapies.

The minimal invasive and easy to use approach of pCNV detection by liquid biopsy would open the possibility to follow the dynamic changes of HER2 expression. This would have important implications for anti-HER2 treatments and open the way to detecting residual resistance and avoid the burden on patients during tissue biopsy.

## Data availability statement

The data associated with the study has been included in the supplementary material file provided with the manuscript.

## CRediT authorship contribution statement

**Susanne Klein-Scory:** Conceptualization, Funding acquisition, Writing – original draft, Writing – review & editing. **Swetlana Ladigan-Badura:** Conceptualization, Data curation, Writing – original draft, Writing – review & editing. **Thomas Mika:** Writing – review & editing. **Berlinda Verdoodt:** Resources, Writing – review & editing. **Andrea Tannapfel:** Resources, Writing – review & editing. **Michael Pohl:** Writing – review & editing. **Roland Schroers:** Supervision, Writing – review & editing. **Alexander Baraniskin:** Conceptualization, Funding acquisition, Investigation, Writing – original draft, Writing – review & editing.

## Declaration of competing interest

The authors declare that they have no known competing financial interests or personal relationships that could have appeared to influence the work reported in this paper.
