# Supplementary material for: Liquid biopsy based HER2 amplification status in gastric cancer patients indicates clinical response
Source: Heliyon. 2023 Nov 2;9(11):e21339. doi: 10.1016/j.heliyon.2023.e21339 (PMC10665680; doi:10.1016/j.heliyon.2023.e21339)
Supplement: Multimedia component 2 [file mmc2.pdf]

Liquid biopsy based *HER2* amplification status in gastric cancer patients indicates clinical response

Figure S1: ddPCR Assay specification

A Details: *HER2* gene FAM-labeled detection assay purchased from Bio-Rad was performed in combination with HEX-labeled reference gene detection assays. The copy number of the target gene HER2 is determined by calculation of the ratio of the HER2 concentration (copies/µl) to the reference concentration (copies/µl), times the number of copies of reference in the human genome: CNV = concentration HER2 (FAM labeled)/ concentration references (Hex labeled) *2. The concentration of HER2 was determined 4-5 fold in combination with the respective reference gene.

Each ddPCR assays was done in a maximal volume of 20 µl per measurement according to manufacturer´s recommendations with 10 µl 2x ddPCR supermix, 1 µl 10x HER2-FAM assay, 1µl 10x Ref-HEX assay, 5 µl DNA sample and 3 µl DNA free water. If gDNA samples were measured, HAEIII restriction enzyme (5 U) was added to the ddPCR mastermix and the volume of aqua was reduced accordingly.

The thermal cycling conditions were 95°C 10 min (1 cycle), 95°C 30 s and 60°C 60 s (40 cycles), 98°C 10 min (1 cycle), and 12°C hold.

Four reference genes were used as reference genes located outside of the chromosome 17 and the genes *EFTUD2* (and TAOK1) as reference genes located near the *HER2* gene on chromosome 17.

B MIQE information about ddPCR assays

Name – assay id validated	Amplicon length	Entrez Gene ID	MIQE context Sequence
**FAM labeled**			
**HER2 alias ERBB2** – dHsaCP1000116	66 nt	2064	hg19|**chr17**:37879834-37879956:+ CGCAGATGCGGATCCTGAAAGAGACGGAGCTGAG GAAGGTGAAGGTGCTTGGATCTGGCGCTTTTGGCA CAGTCTACAAGGTCAGGGCCAGGTCCTGGGGTGG GCGGCCCCAGAGGATGGGGG
Ref 6. TAOK1 dHsaCNS56146077 4	55	57551	hg19|**chr17**:27717972-27718094:+ AGGGTAGCGGCTACCGGAGCGCTGCAGGGGGCTG CGCCTGCCTGCTCCGCCCCAGACCTGTCGGCGAAA GGGTAAGGGCACCTCTGCTTTGGGAAAGGGGGAT ATAGAGGGGGGCGGGGAAAT
HEX labeled			
Ref1. RPP30 – dHsaCP2500350	67	10556	hg19|**chr10**:92660373-92660495:+ TCGGCCATCAGAAGGAGATGAAGATTGTCTTCCAG CTTCCAAGAAAGCCAAGTGTGAGGGCTGAAAAGA ATGCCCCAGTCTCTGTCAGCACTCCCTTCTTCCCTTT TATAGTTCATCAGCCAC
Ref 2. EIF2C1 alias AGO1 dHsaCP2500349	69	26523	hg19|**chr1**:36359312-36359434:+ GAGGGCTACTACCACCCGCTGGGGGGTGGGCGCG AGGTCTGGTTCGGCTTTCACCAGTCTGTGCGCCCTG

			CCATGTGGAAGATGATGCTCAACATTGATGGTGAG TGGGGAGAGCTATGGAGC
Ref 3. TERT dHsaCP2500351	70	7015	hg19|**chr5**:1282570-1282692:+ CCAGACACTCTTCCGGTAGAAAAAGAGCCTGTTCTT TTGAAACGTGGTCTCCGTGACATAAAAGAAAGACC TGAGCAGCTCGACGACGTACACACTCATCAGCCAG TGCAGGAACTTGGCCAG
Ref 4. RPPH1 dHsaCNS674780718	73	85495	hg19|**chr14**:20811218-20811340:+ TTTTTTTCCAAAAATGGGCGGAGGAGAGTAGTCTG AATTGGGTTATGAGGTCCCCTGCGGGGTACCTCAC CTCAGCCATTGAACTCACTTCGCTGGCCGTGAGTCT GTTCCAAGCTCCGGCAA
Ref 5. EFTUD2 dHsaCNS561460774	65	9343	hg19|**chr17**:42927853-42927975:+ CTCTTTTTCACTGGGGGAGAACAGAGTAAGGGACT GGTGGTAGCTGGGGAGAGGACTTGGAGTAAATGG CTGGAAATCAAAGTGCTCTGGCCCCCTACTCCAGG GCAAGGAAGATTCTTAGGG

C Illustration of gene loci on chromosome 17 to discriminate between HER2 Amplification and polysomy of chromosome 17.

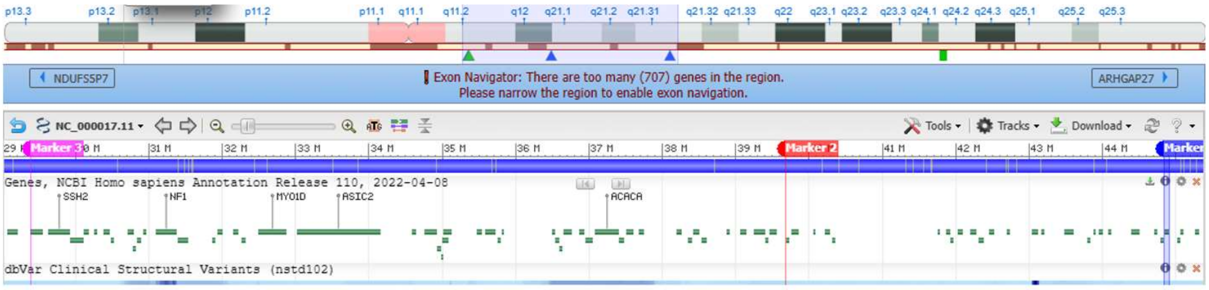

TAOK1

ERBB2
HER2/neu

EFTUD2
