# Supplementary material for: Liquid biopsy based HER2 amplification status in gastric cancer patients indicates clinical response
Source: Heliyon. 2023 Nov 2;9(11):e21339. doi: 10.1016/j.heliyon.2023.e21339 (PMC10665680; doi:10.1016/j.heliyon.2023.e21339)
Supplement: Multimedia component 3 [file mmc3.pdf]

Figure S2 Description of CNV HER2 determination

A Examples of ddPCR analysis of HER2 FAM-labeled assay versus RPP30 (left) or TERT (right) HEX-labeled assay

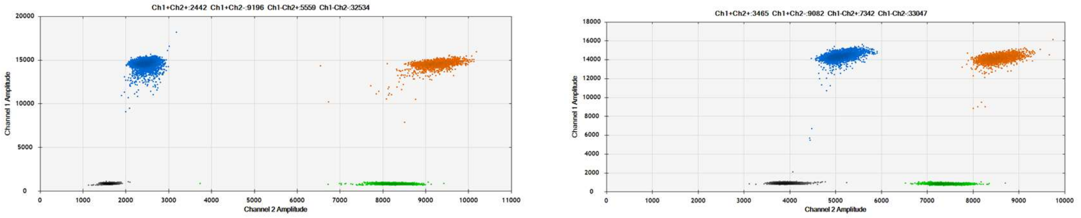

B Examples of the CNV determination of gDNA from cell lines

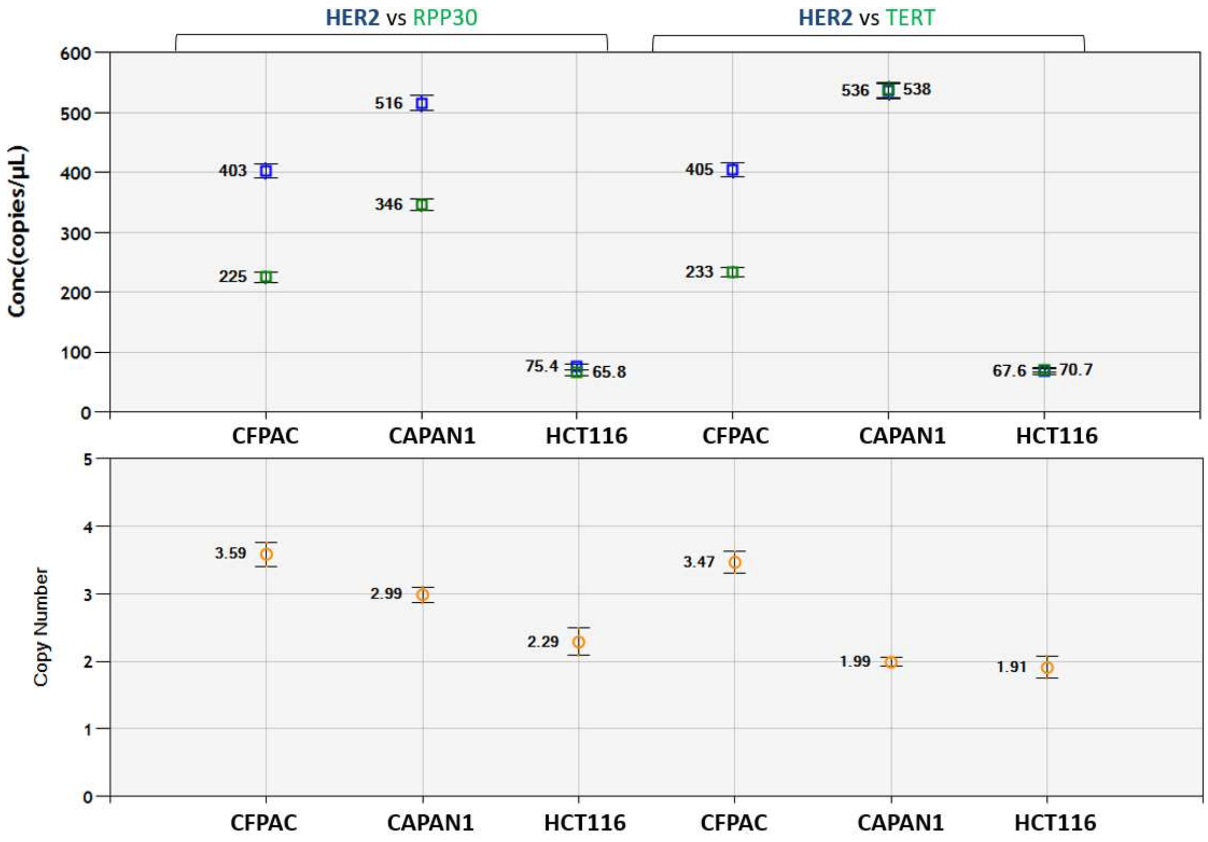

The concentration (Cop/µl) measured in gDNA of cell lines varied individually between reference genes; Copy number variation HER2 calculated by the ratio of HER2 concentration versus reference concentration. Identities of cell lines were confirmed by STR analyses. Error bars indicate 95% confidence interval.
