# Supplementary material for: Liquid biopsy based HER2 amplification status in gastric cancer patients indicates clinical response
Source: Heliyon. 2023 Nov 2;9(11):e21339. doi: 10.1016/j.heliyon.2023.e21339 (PMC10665680; doi:10.1016/j.heliyon.2023.e21339)

Figure S3 Cell lines derived gDNA measurements qualified ddPCR assays for CNV HER2 Determination

A Cosmic data of cell lines compared to ddPCR CNV. The CNV were calculated using the copy numbers of HER2 gene and reference genes annotated in database COSMIC in the cell line project. In comparison, the concentration of HER2 and of reference gene copies were measured by ddPCR assays and the HER2 CNV calculated. Error bars indicate standard deviations.

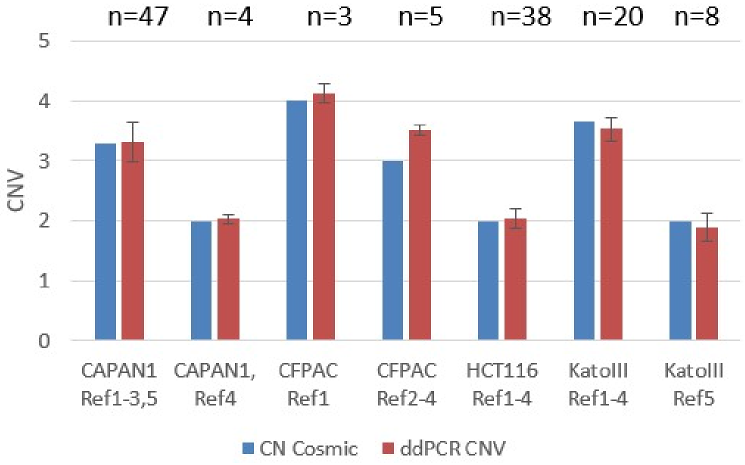

B Accuracy of the ddPCR measurements: Standardization of the ddPCR CNV values measured by different assays indicated that 99.3 % of the values lie within the interval of two standard deviations. z-value = (measured CNV_{assay} – mean CNV_{assay})/standard $deviation_{assay}$)

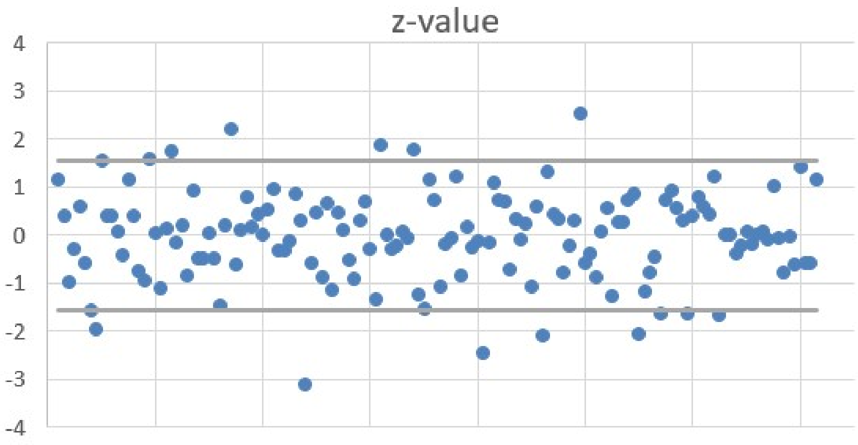

Supplement: Multimedia component 4 [file mmc4.pdf]
