# Supplementary material for: Liquid biopsy based HER2 amplification status in gastric cancer patients indicates clinical response
Source: Heliyon. 2023 Nov 2;9(11):e21339. doi: 10.1016/j.heliyon.2023.e21339 (PMC10665680; doi:10.1016/j.heliyon.2023.e21339)
Supplement: Multimedia component 5 [file mmc5.pdf]

Figure S4 Comparisons between groups regarding CNV results

A Comparison IHC/FISH versus ddPCR tCNV mean by contingency table and Fisher´s exact test

**Data analyzed<br>Cancer patients**	**tissue<br>HER2 pos**	**tissue<br>HER2 neg**	**Total**
**tCNV mean ddPCR >2.4**	10	0	10
**tCNV mean ddPCR <=2.4**	1	6	7
**Total**	11	6	17

P value	0.0006		
P value summary	***		
One- or two-sided	Two-sided		
Statistically significant (P < 0.05)?	Yes		
		CI 95%	
Sensitivity	0,9091	0,6226 to 0.9953	
Specificity	1	0.6457 to 1.000	
Positive Predictive Value	1	0.7225 to 1.000	
Negative Predictive Value	0.875	0.5291 to 0.9936	
Likelihood ratio	*LR+ 2.8*	*LR- 0.377*	

B Comparison of tCNVs in relation to different reference genes by Spearman correlation

**variant A**	**variant B**	**n**	**spearman coefficient**	**assessment**
tCNV ref1 RPP30	tCNV ref2 EIF2C1	11	0.98	strong
	tCNV ref3 RPPH1	11	0.98	strong
	tCNV ref5 EFTUD2	11	0.88	strong
tCNV ref2 EIF1C1	tCNV ref3 RPPH1	11	0.96	strong
	tCNV ref5 EFTUD2	11	0.90	strong
tCNV ref3 RPPH1	tCNV ref5 EFTUD2	11	0.92	strong
variant A	variant B	n	spearman coefficient	assessment
IHC/FISH	tCNV mean	11	0.417	moderate
IHC/FISH	tCNV ref1 RPP30	11	0.351	moderate
IHC/FISH	tCNV ref2 EIF2C1	11	0.431	moderate
IHC/FISH	tCNV ref3 RPPH1	11	0.418	moderate
IHC/FISH	tCNV ref5 EFTUD2	11	0.499	moderate

C pCNV ddPCR compared to HER2 Status by IHC/FISH

Data analyzed<br>Cancer patients	*HER2<br>pos*	*HER2<br>neg*	*Total*
pCNV ddPCR >2.4	2	0	2
pCNV ddPCR <=2.4	8	7	15
Total	10	7	17

Test	Fisher's exact test	
P value	0.4853	
P value summary	ns	
One- or two-sided	Two-sided	
Statistically significant (P < 0.05)?	No	
Sensitivity	0.2000	0.03554 to 0.5098
Specificity	1.000	0.6457 to 1.000
Positive Predictive Value	1.000	0.1777 to 1.000
Negative Predictive Value	0.4667	0.2481 to 0.6988
Likelihood Ratio	LR+ 20	LR- 0.5

D Comparison of ddPCR based pCNV and HER2 status

Contingency table s including GC patients HER2 positive, GC patients HER2 negative and non-cancer patients

**mean**	**GC pos**	**GC neg+ non cancer H**
ddPCR < 2.4	2	0
ddPCR <= 2.4	10	29
rpp30	**GC pos**	**GC neg+ non cancer H**
ddPCR < 2.4	3	0
ddPCR <= 2.4	9	21
eif2C1	**GC pos**	**GC neg+ non cancer H**
ddPCR < 2.4	3	2
ddPCR <= 2.4	9	29
Eftud2	**GC pos**	**GC neg+ non cancer H**
ddPCR < 2.4	3	0
ddPCR <= 2.4	9	28
tert	**GC pos**	**GC neg+ non cancer H**
ddPCR < 2.4	1	1
ddPCR <= 2.4	7	15
rpph1	**GC pos**	**GC neg+ non cancer H**
ddPCR < 2.4	2	8
ddPCR <= 2.4	5	20

Results of contingency tables

**Comparison Fisher´s exact test Cut off 2.4**	P-value (α 0.05)	Sensitivity	Specificity	Positive Predictive Value	Negative Predictive Value	Likelihood Ratio
**pCNV Mean 4-5 reference genes**	**0.0242***	0.17	1.00	1.00	0.74	16.67
Ci 95%		0.029 to 0.448	0.88 to 1.000	0.18 to 1.000	0.59 to 0.85	
**pCNV Ref5 (EFTUD2)**	**0.0223***	0.25	1.00	1.00	0.76	25.00
Ci 95%		0.089 to 0.53	0.88 to 1.000	0.44 to 1.000	0.59 to 0.87	
**pCNV Ref1 (RPP30)**	**0.0403***	0.25	1.00	1.00	0.70	25

Ci 95%		0.089 to 0.53	0.84 to 1.00	0.44 to 1.00	0.52 to 0.83	
pCNV Ref2 (EIF2C1)	0.123	0.25	0.94	0.60	0.76	3.875
Ci 95%		0.089 to 0.53	0.79 to 0.99	0.23 to 0.93	0.61to 0.87	
pCNV Ref3 (TERT)	>0.9999	0.25	1.00	1.00	0.76	2
Ci 95%		0.08 to 0.53	0.88 to 1.00	0.44 to 1.00	0.59 to 0.87	
pCNV Ref4 (RPPH1)	>0.9999	0.29	0.71	0.20	0.80	1
Ci 95%		0.05 to 0.64	0.53 to 0.85	0.035 to 0.51	0.61 to 0.91	
