# Supplementary material for: Liquid biopsy based HER2 amplification status in gastric cancer patients indicates clinical response
Source: Heliyon. 2023 Nov 2;9(11):e21339. doi: 10.1016/j.heliyon.2023.e21339 (PMC10665680; doi:10.1016/j.heliyon.2023.e21339)
Supplement: Multimedia component 6 [file mmc6.pdf]

Figure S5 CNV HER2 of blood gDNA and CNV HER2 determined from ctDNA

CNV HER2 of blood cell gDNA (light grey) and CNV HER2 of ctDNA from plasma (pCNV. dark grey) of gastric cancer patients were determined from corresponding blood collection samples. The haematopoeitic gDNA (cell CNV) value can be used as control of the ctDNA based CNV determination. This make it possible to verify that the increased HER2-CNV levels are not due to genomic changes in hematopoiesis.

After centrifugation of whole blood and the careful removal of plasma the gDNA of blood cells were isolated from 200 µl cellular fraction using the Qiagen Blood gDNA kit. The gDNA used in the ddPCR assay as described in methods section. In brief 5 µl of the eluates were added to 15 µl ddPCR mastermix containing the particular HER2 and reference assays.

A

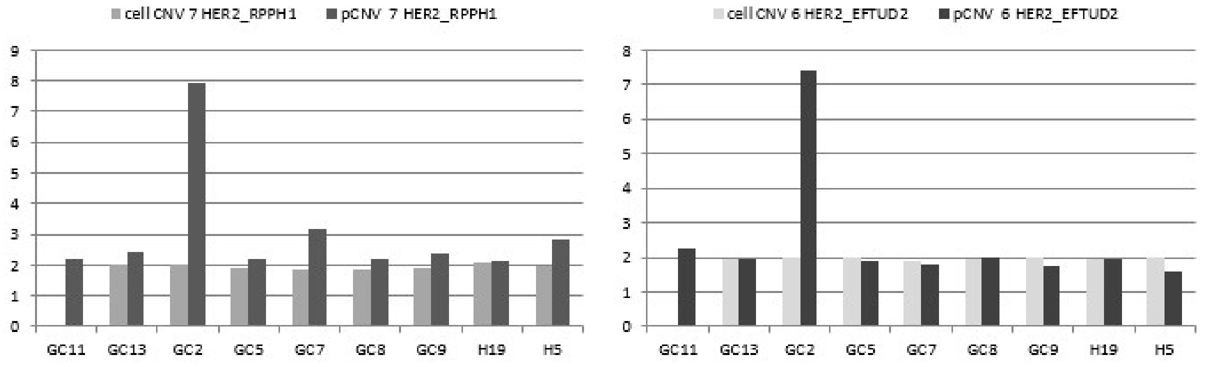

B

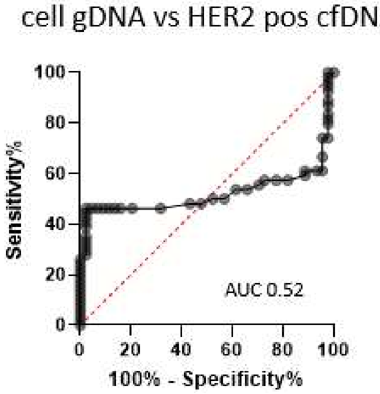

	Sensitivity%	95% CI	Specificity%	95% CI	Likelihood ratio
> 2.365	29,63	19,14% to 42,83%	97,73	88,19% to 99,88%	13,04
> 2.375	27,78	17,62% to 40,89%	97,73	88,19% to 99,88%	12,22
> 2.390	25,93	16,12% to 38,93%	100,0	91,97% to 100,0%	
> 2.410	24,07	14,64% to 36,95%	100,0	91,97% to 100,0%	
