# Supplementary material for: Liquid biopsy based HER2 amplification status in gastric cancer patients indicates clinical response
Source: Heliyon. 2023 Nov 2;9(11):e21339. doi: 10.1016/j.heliyon.2023.e21339 (PMC10665680; doi:10.1016/j.heliyon.2023.e21339)

Figure S6

HER2 amount in ctDNA samples. The absolute amount of HER2 copies in plasma summarized for patients´group was shown in respect to the blood collection time points (x-axis). Most blood collections took place during treatment (blue arrow).

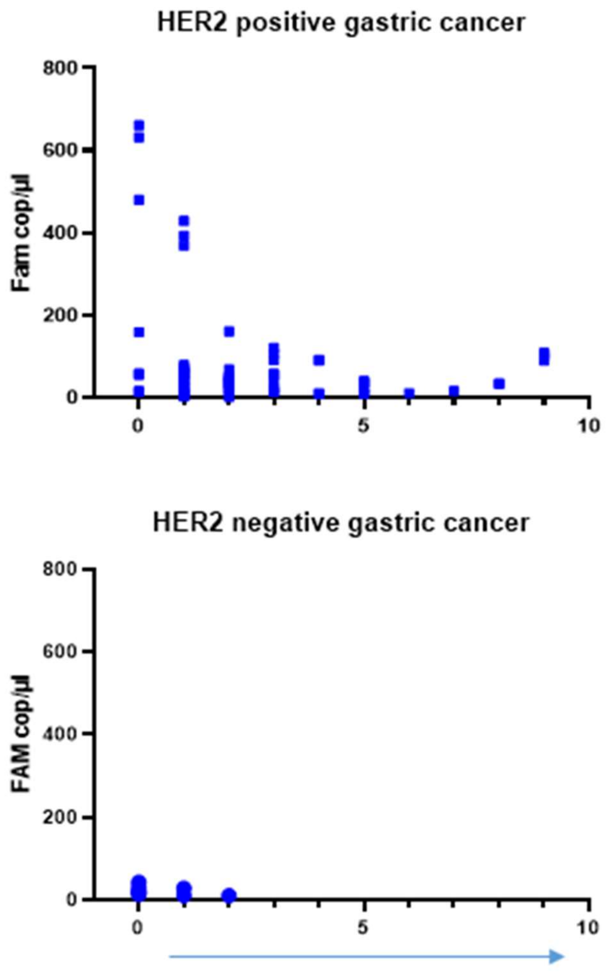

Supplement: Multimedia component 7 [file mmc7.pdf]
