# Supplementary material for: Liquid biopsy based HER2 amplification status in gastric cancer patients indicates clinical response
Source: Heliyon. 2023 Nov 2;9(11):e21339. doi: 10.1016/j.heliyon.2023.e21339 (PMC10665680; doi:10.1016/j.heliyon.2023.e21339)
Supplement: Multimedia component 8 [file mmc8.pdf]

Additional Information to “*Liquid biopsy based HER2 status in gastric cancer*”

Figure S7 Data summery and gastric cancer characteristics

Patients and number of samples summary

Patient group by tissue based HER2 amplification status	#patients	Tissue analysis	#plasma samples	#cellDNA blood	Measurements ctDNA HER2 vs reference genes
HER2 status tissue based HER2 positive	12	11	34	12	215
HER2 Status tissue based HER2 negativ	7	0	13	1	65
Non cancer HER2 status not determined	22	0	25	2	135
all	42	11	72	15	462
